# Concordance of Death Information Between Two Health Systems Serving the Same Region and the Social Security Administration Death Master File: Retrospective Observational Study

**DOI:** 10.2196/85136

**Published:** 2026-04-07

**Authors:** Zhan Wang, Mahanaz Syed, Shweta Bansal, Meredith Nahm Zozus, Eric Eisenstein, Muayad Hamidi

**Affiliations:** 1 Clinical Research Informatics Division, Department of Population Health Sciences, University of Texas Health Science Center at San Antonio, 7703 Floyd Curl Drive, San Antonio, TX, 78229, United States, 1 2105624107; 2 Division of Nephrology, Department of Medicine, University of Texas Health Science Center at San Antonio, , 7703 Floyd Curl DriveSan Antonio, TX, 78229, United States; 3 Department of Medicine, Duke University, Durham, NC, United States; 4 School of Health Information Science, University of Victoria, Victoria BC, CN, Canada

**Keywords:** death record concordance, health systems, mortality data quality, electronic health record data quality, EHR data quality, Social Security Administration Death Master File

## Abstract

**Background:**

There are multiple sources that document the date of a person’s death in the United States. Unfortunately, this seemingly simple information is either incomplete or costly to obtain. Nevertheless, information on such events is critical for both health systems and clinical studies to assess the outcomes of operational and therapeutic interventions.

**Objective:**

As part of a larger assessment of the quality of multisource death information, we compared the death data from two health systems serving the same region and the Social Security Administration Death Master File (SSADMF).

**Methods:**

This study linked death records for patients seen in either health system with the SSADMF from 2007 to 2020 to identify concordant and discordant death data among sources. Analyses included cross-system matching, classification of death records by overlap, and calculation of agreement using the Fleiss κ.

**Results:**

Among 904,581 matched patients, only 209 (0.02%) deaths were confirmed by all 3 sources. Large proportions of deaths were uniquely recorded by a single source: 54.32% (10,697/19,691) by health system A, 5.16% (1017/19,691) by health system B, and 20.17% (3972/19,691) by the SSADMF. The Fleiss κ was negative (−0.312), reflecting less agreement than expected by chance.

**Conclusions:**

While this study is not generalizable, it showed that, without processes in place to obtain external data regarding patient deaths, health care facility death information should not be relied upon as a complete list of those who have died. The discordances observed highlight the potential for significant gaps in death reporting within health care systems, which could impact the accuracy of mortality-based analyses and quality assessments.

## Introduction

Mortality is the ultimate outcome of health care and wellness interventions. As such, mortality data are important to many clinical studies. In the era of learning health systems and real-world evidence, institutions increasingly rely on routinely collected electronic health record (EHR) data to evaluate care delivery, monitor outcomes, and iteratively improve practice [1,2]. However, the accuracy and completeness of mortality information within these data remain uncertain, particularly for deaths that occur outside individual health systems [3,4].

Our clinical data warehouse, serving a large academic health center in the southwestern United States, is used for approximately 100 clinical studies per year. Many of these studies focus on our local population and address whether they improve or worsen particular outcomes and include tracking or intervening in health disparities in our region, including observational comparative effectiveness studies, local quality improvement initiatives, and program evaluations targeting population health and health disparities in the surrounding region. These data are used primarily for retrospective and hybrid retrospective-prospective designs, where mortality end points are often ascertained from EHRs and linked administrative sources rather than from dedicated, trial-specific follow-up. Because findings from such studies can inform guideline development, institutional policies, and clinical decision support within learning health systems, inaccurate or incomplete mortality data can directly affect patient care.

In clinical research evaluating treatments for diseases with low mortality rates (eg, due to short follow-up), errors in the few available death data points can introduce significant bias, potentially compromising the validity of the study [5]. Clinical studies are increasingly using EHR real-world data in the United States, where recent regulatory guidance requires assessment of accuracy and completeness [1]. Unfortunately, many institutions lack comprehensive information about death for patients who die outside of their facilities, making the use of EHR real-world data less reliable in these studies. The increasing trend of in-home end-of-life care further limits mortality data in EHRs [6].

Researchers and clinicians in the United States have used public data sources such as the National Death Index (NDI) or the Social Security Administration Death Master File (SSADMF) to obtain mortality data [7-10]. The reports of NDI sensitivity range from 87% to 97%, with SSADMF sensitivity ranging from 71% to 83%, although performance varies by cohort, identifiers available, and period [3,11-15]. Importantly, a Social Security Administration policy change in November 2011 removed protected state death records from the publicly disseminated Death Master File, eliminating approximately 4.2 million existing records and sharply reducing capture of more recent deaths, with continuing implications for the completeness of SSADMF-based mortality ascertainment [16]. These national resources; near ubiquitous EHR adoption; and significant strides in EHR interoperability, such as health information exchanges supporting the long-used Health Level Seven Admission, Discharge, and Transfer message that can indicate a death, imply good availability of reliable death information. However, obtaining high-quality data for mortality end points in clinical studies remains a significant challenge [17,18].

These national sources are also costly and logistically constrained. The costs of obtaining external death information range from 15 cents per patient per year (NDI) to an approximately US $3000 initial fee and US $7000 annual subscription fee per user for the Limited Access SSADMF [4]. The NDI data are only available for research, their use is time limited, and reuse is constrained through a data use agreement. These limitations lead some health systems to purchase and routinely integrate such data and others to rely solely on local or regional sources [3].

Even when national mortality data are purchased and linked, there is no single contemporary gold standard for deaths occurring outside a given health system, and both national and local sources show imperfect sensitivity and date accuracy [19]. Increasing trends toward in-home and hospice end-of-life care further reduce the proportion of deaths directly captured within hospital EHR systems [20]. Consequently, one can reasonably argue that all health systems lack fully comprehensive information about out-of-hospital deaths, and that practical strategies rely on combining multiple imperfect data sources to approximate a gold standard for mortality ascertainment.

Academic health centers need a sustainable way to maintain high-quality mortality data linked to health care data. For example, our cancer registry requires death information, as do many clinical trials. In this context, we posed two major questions. (1) What is the accuracy of death data, such as occurrence and date? (2) What is the quality and relative contribution from available death data sources? To begin, we compared patient deaths reported in our health system, a neighboring health system serving the same region, and the SSADMF.

## Methods

### Data Sources and Linkage

This study evaluated the concordance of death information between 2 health systems serving the same geographic region and the SSADMF over a 13-year period (January 1, 2007, to July 10, 2020), as determined by the availability of data from both health systems. Patient records from the 2 health systems were linked using three approaches: (1) an existing identifier crosswalk; (2) matching first name, last name, gender, and birth date; and (3) matching medical record number. To link SSADMF data with the health system records, five distinct hash-based tokens were used: (1) last name, first initial of first name, gender, and birth date; (2) Soundex last name, Soundex first name, gender, and birth date; (3) last name, first name, gender, and birth date; (4) Social Security number, gender, and birth date; and (5) Social Security number and first name. For linkage, a match on both token 1 and token 2 was required, whereas tokens 3, 4, and 5 were each sufficient for linkage when matched individually.

### Death Record Categorization

Death records were classified into mutually exclusive groups to capture concordance and discordance across the 3 sources. For the matched patient cohort (patients identified in both health systems), deaths were categorized as follows: (1) concordant deaths were deaths recorded in all 3 sources (health system A, health system B, and the SSADMF), and (2) discordant deaths were deaths recorded in only 1 or 2 sources among the matched patients, further subdivided into those recorded only in health system A, recorded only in health system B, recorded only in the SSADMF, and recorded in combinations of 2 sources (eg, health systems A and B, health system A and the SSADMF, or health system B and the SSADMF). For patients present in a single health system, deaths recorded exclusively in that system or in the SSADMF were also identified. This approach allowed us to distinguish between deaths that were consistently captured across all sources and those that were discordant, reflecting differences in reporting or population coverage.

To further investigate discordant death records, we analyzed “lost contact” patients, defined as individuals who had at least 1 encounter at 1 facility during the observation period (January 1, 2007, to July 10, 2020) but no subsequent encounters at that facility for at least 12 consecutive months after their last visit there while maintaining contact with the other facility after those 12 months. This analysis helped identify potential gaps in follow-up or differences in patient retention between systems.

In addition, we performed 2 exploratory analyses: date discrepancies of more than 1 month for deaths recorded between sources and postdeath encounters occurring more than 1 year after the recorded death date. The first analysis indicated inconsistencies in death reporting between sources, which can affect the accuracy of mortality statistics and clinical research. For postdeath encounters, we compared the death dates from the 3 sources to the encounter dates from both health systems. The analysis revealed potential issues in death recording or patient management, suggesting inaccuracies in encounter or death records or possible misuse of deceased individuals’ health care credentials.

### κ Analysis for Intersource Agreement

To measure the consistency of death reporting across the 3 data sources, we calculated observed agreement; expected agreement; and the Fleiss κ, a statistic used to evaluate agreement among multiple raters with categorical data. Each patient in the matched cohort was classified as either “dead” or “not dead” by each source. We then grouped patients into 8 mutually exclusive categories based on the combination of sources that reported their death. These metrics allowed us to assess the level of concordance in death classification across health system A, health system B, and the SSADMF.

### Visualization

A 3-set Venn diagram was constructed to illustrate the overlap and unique contributions of each data source in identifying deaths within the matched patient cohort. Each region of the diagram was annotated with the corresponding patient counts, providing a comprehensive view of concordance and discordance in death ascertainment across health system A, health system B, and the SSADMF. This visualization facilitated the identification of patterns and gaps in death reporting among the 3 sources.

### Ethical Considerations

This study was reviewed by the Institutional Review Board of the University of Texas Health Science Center at San Antonio and determined to meet the criteria for exemption from institutional review board review in accordance with Title 45 of the Code of Federal Regulations §46.104 [21] on February 21, 2024 (STUDY00000154). As this research involved secondary analysis of existing death data and no direct interaction with human participants, informed consent was not required. All data were handled in accordance with institutional and national ethical standards to ensure the privacy and confidentiality of the individuals’ information.

## Results

**Figure 1.**
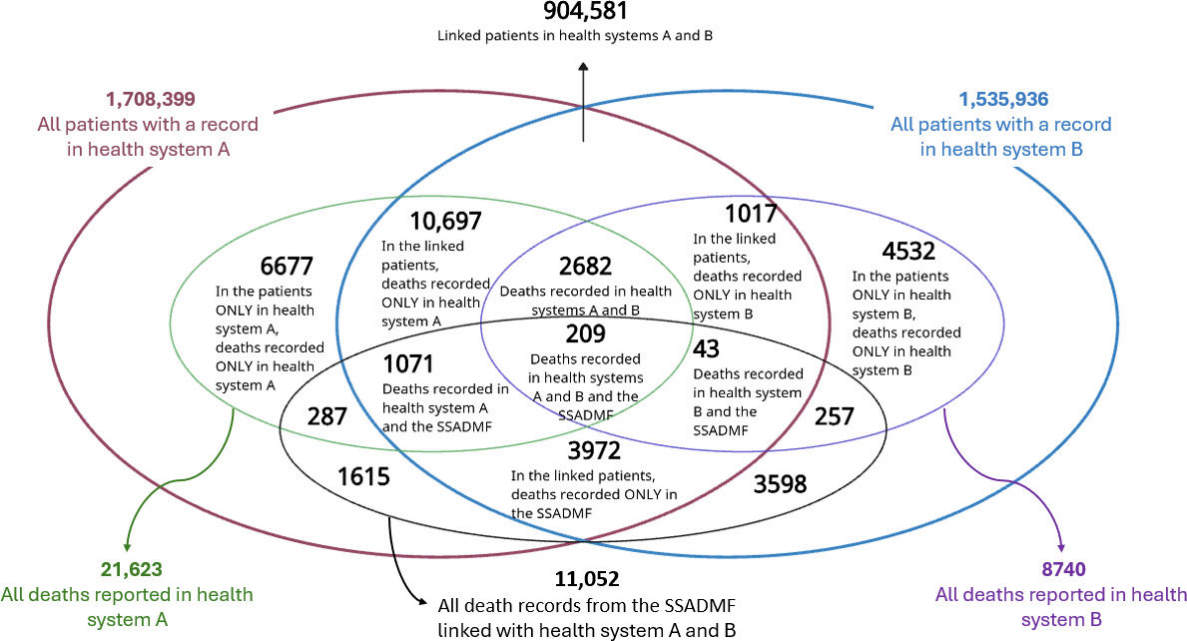
Patients and deaths reported among health systems A and B and the Social Security Administration Death Master File (SSADMF; encompassing matched and unmatched patient records).

Health system A reported 1,708,399 unique patients, including 21,623 (1.27%) reported deaths, while health system B reported 1,535,936 unique patients, including 8740 (0.57%) reported deaths (Figure 1). A total of 11,052 death records from the SSADMF were linked to the combined cohort, encompassing both matched and unmatched patient records from the 2 health systems.

**Figure 2.**
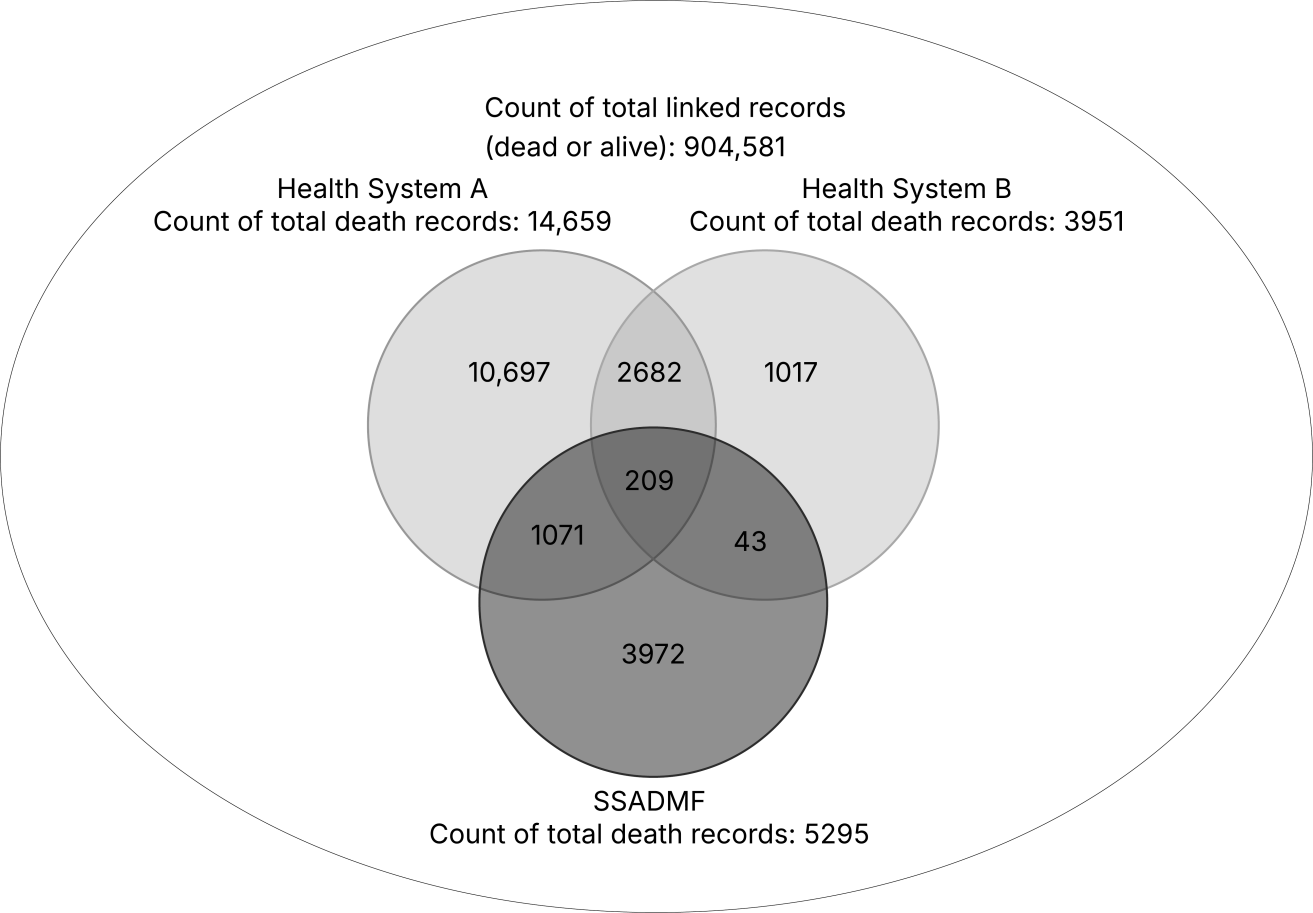
Three-set Venn diagram for the matched patient records. SSADMF: Social Security Administration Death Master File.

Among the 904,581 matched patients, health system A reported 14,659 (1.62%) deaths, health system B reported 3951 (0.44%) deaths, and the SSADMF identified 5295 (0.59%) deaths (Figure 2). Collectively, the total number of unique death records across these sources was 19,691. Figure 2 shows that 1.06% (209/19,691) of deaths were recorded by all 3 sources, whereas 54.32% (10,697/19,691) of deaths were identified exclusively by health system A, 5.16% (1017/19,691) were identified exclusively by health system B, and 20.17% (3972/19,691) were identified exclusively by the SSADMF. The proportion of death overlap between health system A and the SSADMF, health system B and the SSADMF, and the 2 health systems was 5.44% (1071/19,691), 0.22% (43/19,691), and 13.62% (2682/19,691), respectively. Health system A contributed 74.45% (14,659/19,691) of the unique deaths observed in at least one source, health system B contributed 20.07% (3951/19,691), and the SSADMF contributed 26.89% (5295/19,691).

To quantify overall agreement in death classification among the 3 sources, the Fleiss κ was calculated using the distribution of death status assignments across all matched patients (Table 1). The observed agreement, defined as the proportion of patients for whom all 3 sources concurred on death status (either all “dead” or all “not dead”), was 0.9771. The expected agreement by chance based on the marginal probabilities of each source assigning “dead” or “not dead” was 0.9826. The Fleiss κ, summarizing agreement beyond chance, was −0.312, with a 95% CI from −0.330 to −0.294.

**Table 1.** Distribution of death status among the 3 sources in the matched cohort (N=904,581).

Overlap category	Patients, n (%)
Deaths in all 3 sources	209 (0.02)
Deaths in health systems A and B only	2682 (0.30)
Deaths in health system A and the SSADMF^a^ only	1071 (0.12)
Deaths in health system B and the SSADMF only	43 (0.00)
Deaths in health system A only	10,697 (1.18)
Deaths in health system B only	1017 (0.11)
Deaths in the SSADMF only	3972 (0.44)
Not dead in any source	884,890 (97.82)

a SSADMF: Social Security Administration Death Master File.

Further investigation of discrepancies in matched patients revealed that, of the 11,768 patients recorded as deceased in health system A but not in health system B, 4796 (40.75%) lost-contact patients in health system B switched to health system A. Similarly, of the 1060 patients recorded as deceased in health system B but not in health system A, 44 (4.15%) lost-contact patients in health system A switched to health system B. These lost-contact patterns suggest that patient migration between facilities may contribute to the discordance in death reporting.

To explore the potential benefit of data quality checking rules, we identified 0.20% (39/19,691) of cases in which the date of death recorded in health system A differed by 1 month or more (median 2, range 1 to 20 months) from that recorded in health system B. Similarly, 0.23% (45/19,691) of the cases showed a discrepancy of at least 1 month (median 29; range 1-122 months) between the date of death recorded in health system A and the SSADMF, whereas 0.02% (3/19,691) of the cases showed a discrepancy of at least 1 month (median 52; range 49-122 months) between the date of death recorded in health system B and the SSADMF.

An analysis of postdeath encounters revealed that 1.22% (179/14,659) of the cases from health system A had visits recorded in either of the 2 health systems more than 1 year following their documented date of death. Similarly, 0.35% (14/3951) of such cases were identified in health system B, and 8.48% (449/5295) of cases were identified within the SSADMF.

## Discussion

### Principal Findings

Death records from 2 major health systems serving the same region and the SSADMF provide new insights into the complexities and limitations of mortality reporting in health care research. Ideally, each health system would maintain a complete and accurate record of all deaths, including precise date of death for every deceased patient. However, our study revealed substantial variability and discordance in mortality ascertainment across these 3 sources, underscoring the challenges inherent in relying solely on health care facility records for vital status determination.

Significant discrepancies were observed in both the identification of deaths and the recording of death dates among the 2 health systems and the SSADMF. The Venn diagram analysis demonstrated that a considerable number of deaths were uniquely captured by only 1 data source, with relatively few cases identified by all 3. This pattern highlights the risk of systematic bias and under-ascertainment when mortality surveillance depends on a single institutional record system. The presence of deaths recorded exclusively by the SSADMF (deaths not captured in either health system) emphasizes the value of integrating external sources to enhance completeness.

In Texas, hospitals are required to report deaths that occur within their facilities to the local registrar, who then forwards this information to the Texas Department of State Health Services Vital Statistics Section. This process ensures that deaths occurring in hospitals are captured in the official state vital records. However, a critical regulatory change further complicates the concordance between health system records and the SSADMF. Deaths reported solely to state vital record offices after November 1, 2011, may not appear in the publicly available SSADMF even if they are accurately captured in hospital or state databases. These reporting practices and regulatory constraints may explain the observed discrepancies between the health system data and the SSADMF. Deaths that occur in hospitals and are reported to the state may be present in the state’s vital records but absent from the SSADMF if they are considered protected state records. Conversely, deaths reported directly to the Social Security Administration through other means (such as funeral directors or family members) may appear in the SSADMF but not in hospital records if the patient was not under active care at the time of death or if the hospital was not notified.

Analysis of lost-contact patients suggests that the discrepancies in death reporting may be partially explained by differences in patient engagement with each health system over time. Patients who continued to receive care primarily from one system or had no recorded visits in the other system were more likely to have their death recorded by the system with which they last interacted, potentially due to more up-to-date information or discovering deaths through postcare follow-up or billing interactions. The higher proportion of health system B–to–health system A switching compared to health system A–to–health system B switching explains part of health system A’s substantially greater volume of death records overall. These findings suggest that patient migration between systems contributes to death reporting discordance, although the reasons for migration require further investigation. While these findings offer insight into the discrepancies, they do not fully account for the substantial differences observed. This substantial difference suggests that additional factors such as institutional practices, death documentation processes, or data management systems may significantly influence death reporting.

The presence of postdeath encounters in both systems is particularly concerning. These instances could represent data entry errors, identity mix-ups, fraudulent use of a deceased patient’s identity and health care coverage by another individual or entity, or other root causes. These should be investigated to identify opportunities for improvement. The results reported in this paper emphasize the importance of cross-referencing death information from multiple sources to identify discrepancies and incompleteness.

The integration of the SSADMF into our analysis allowed for a more robust assessment of concordance across sources. A high observed agreement was driven primarily by the predominance of patients classified as “not dead” by all sources. In the context of this highly imbalanced outcome, the negative κ should not be interpreted as strong paradoxical disagreement but rather as a statistical artifact of extreme class imbalance where chance agreement among “not dead” classifications is very high. Therefore, the Venn diagrams and Table 1 provide a more informative description of how often each source captured deaths and how frequently those deaths were uniquely identified by one source vs shared across sources. The finding is that the 3 sources showed limited concordance in identifying deaths themselves.

### Limitations

A persistent limitation of our study is the absence of a definitive gold standard for mortality ascertainment. Without such a reference, we are unable to calculate accuracy statistics such as sensitivity and specificity and cannot definitively identify false positives or false negatives. While some potential false positives may be inferred from the presence of postdeath encounters, the true extent of missed deaths (false negatives) remains unknown and likely requires external validation. Errors in patient linkage, crosswalks, or record attribution may also contribute to misclassification. In addition, differences in linkage methodology may influence the observed discordance: the SSADMF linkage required agreement on 2 composite name–demographic tokens recommended by the data provider to balance tolerance for name variation, a design that may reduce missed matches but carries a higher risk of false linkages when names are similar yet pertain to different individuals. Future work should incorporate more reliable data sources, including the NDI, state death records, obituary data, and family-reported information. These additional sources would provide a more comprehensive and accurate picture of mortality, allowing for a better assessment of false positives and false negatives in health care facility death data.

We report this evaluation as a marker and example of significant discordance in death data among 2 health systems serving the same region and the SSADMF. While not an unexpected or groundbreaking result, such assessments serve as important reminders that, although we have come a long way toward EHR adoption and interoperability of health information, we have a long way to go before even the most basic health care data can be assumed to be accurate and complete.

### Conclusions

This study demonstrates that death data from health care facilities, even when supplemented with data from the SSADMF, exhibit substantial discordance and should not be presumed to be accurate or complete without corroboration from additional sources. The persistent discrepancies among the 3 sources highlight the limitations and potential unreliability of depending solely on the EHR. Despite advances in EHR adoption and interoperability, obtaining high-quality, comprehensive mortality data for research remains challenging. Researchers and clinicians should not rely solely on facility death data for mortality end points in observational studies unless death information is systematically and prospectively collected as part of the study protocol; instead, they are strongly encouraged to incorporate external sources, such as the NDI or state vital records, to improve accuracy and completeness. Further research is needed to understand the causes of discordance and develop strategies for enhancing the quality of mortality information in clinical and epidemiological studies.

## Abbreviations

EHR: electronic health record
NDI: National Death Index
SSADMF: Social Security Administration Death Master File
